# Alpha-lipoic acid reduces oxidative damage and ameliorates follicular abnormalities in vitrified cat ovarian tissue

**DOI:** 10.3389/fendo.2025.1682526

**Published:** 2025-10-13

**Authors:** Shuyuan Yin, Fenghua Li, Zhennan Pan, Hongchu Bao, Xiaoyan Liu

**Affiliations:** ^1^ Reproductive Medicine Centre, Yantai Yuhuangding Hospital, Qingdao University, Yantai, China; ^2^ Shandong Provincial Key Medical and Health Laboratory of Reproductive Health and Genetics (Yantai Yuhuangding Hospital), Yantai, China

**Keywords:** ovary, vitrification, oxidative damage, follicle development, transcriptome sequencing

## Abstract

**Introduction:**

Vitrification may induce oxidative damage, mitigating cryodamage is crucial for preserving follicular development and ovarian function. Alpha lipoic acid (α-ALA) is an antioxidant which exerts its antioxidant activity directly or indirectly.

**Methods:**

In this study, we established a α-ALA concentration gradients (0, 100, and 150 μM) in the vitrification solution, assessed follicular abnormalities through hematoxylin and eosin (HE), analyzed cell apoptosis via TUNEL staining analysis, immunohistochemistry (IHC) and Masson staining, explored differential genes by transcriptome sequencing.

**Results:**

Our findings revealed that α-ALA alleviated oxidative stress damage and decreased follicular abnormalities induced by vitrification, enhanced antioxidant capacity and delayed the onset of fibrosis during in vitro culture. Moreover, our analysis revealed the effects of α-ALA on vitrification at the transcriptomic level.

**Discussion:**

In this study, we focused on the anti-apoptotic effects of α-ALA in cat ovarian cortex vitrification, and concluded that α-ALA effectively mitigates vitrification correlated ovarian functional impairment.

## Graphical Abstract

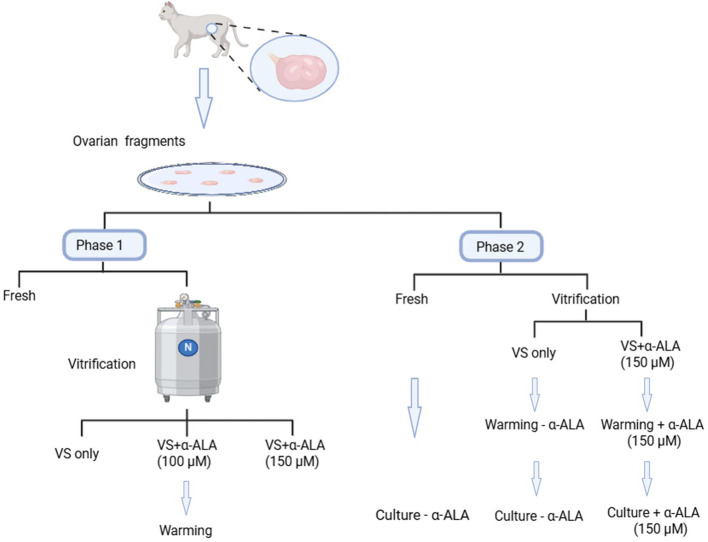

## 1 Introduction

The ovaries are essential reproductive and endocrine organs that secrete hormones, including estrogen and progesterone, which regulate female physiological health (1). The ovarian cortex contains a large number of primordial follicles, and their maturation and ovulation are critical for natural conception (2). Under certain circumstances, ovarian tissue cryopreservation may be required. Ovarian tissue cryopreservation provides unique advantages, however, it also presents several limitations. It is essential to minimize massive follicle loss during freezing and thawing processes, prevent damage to the ovarian stroma, reduce ischemic injury during transplantation, and preserve ovarian viability and function as much as possible.

Ovarian tissue cryopreservation primarily involves slow freezing and vitrification. Slow freezing is predominantly employed and reported to be successful in live births at present. The process involves exposing the tissue to a lower concentration of cryoprotectant, then gradually freezing it to -130°C using a programmed freezing device, and ultimately storing the tissue in liquid nitrogen for long-term preservation. While slow freezing demonstrates the potential for mitigating certain cellular damage through controlled-rate cooling, several limitations still exist. Compared to slow freezing, vitrification is easier and more economical, which can effectively reduce cell damage caused by intracellular ice crystal formation. Vitrification is a freezing method in which tissues are soaked in high concentrations of cryoprotectants and then rapidly transferred into liquid nitrogen to produce a “vitrified state” for preservation. In ovarian tissue vitrification, the cryoprotectants are primarily combined to include permeable and non-permeable ones, thereby reducing damage caused during the process. Permeable cryoprotectants include ethylene glycol, dimethyl sulfoxide (DMSO), propylene glycol, glycerol, and others. Non-permeable cryoprotectants primarily consist of saccharides and polymers, such as sucrose, trehalose, raffinose, polyethylene glycol, and polyvinylpyrrolidone (3, 4). The primary issues associated with vitrification are that high concentrations of cryoprotectants may produce chemical toxicity and osmotic toxicity, which can trigger apoptosis. Reactive oxygen species (ROS) play a dual role in regulating multiple signaling pathways, thereby influencing follicular homeostasis. Physiological levels of ROS support normal follicular development, whereas excessive ROS accumulation induces apoptotic pathways and mediates oxidative damage mechanisms through lipid peroxidation and ferroptosis, ultimately resulting in nucleic acid degradation and nuclear membrane disruption (5). ROS generation has been implicated in the dynamics of primordial follicle activation, with supraphysiological concentrations stimulating crucial signaling cascades, including MAPK, PI3K/AKT/mTOR, NF-κB, and other signaling pathways, thereby promoting primordial follicle activation (6). Mouse ovarian vitrification not only induces apoptosis but also alters gene expression, changes cell membranes and cytoskeletons, and the extracellular matrix, and causes ultrastructural changes in the endoplasmic reticulum (7).

Ovarian vitrification may lead to massive loss and abnormal activation of primordial follicles. Studies indicate that prior to revascularization after ovarian tissue transplantation, over 60% of follicles may undergo atresia, with 80% of primordial follicles becoming activated, resulting in a significant depletion of the primordial follicle pool and affecting ovarian reserve (8). As the primordial follicle pool is non-renewable, both follicle atresia and large-scale activation can contribute to premature ovarian failure. Researchers added antioxidants during vitrification and/or thawing processes to reduce damage caused by vitrification. α-ALA is an antioxidant that can exert its effects by directly scavenging ROS, in the other hand, it also exerts its antioxidant activity indirectly, such as contributing to the regeneration of glutathione and vitamin C, participating in mitochondrial metabolism, and reducing mitochondrial oxidative stress. α-ALA has been indicated to ameliorate oxidative lipid damage and lipoapoptosis induced by charged oxygen ion radiation in mouse ovaries, partially alleviating oxidative damage in granulosa cells and oocytes (9). Studies have reported that supplementation with α-ALA during ovine ovarian vitrification increased intracellular glutathione levels, thereby eliminating ROS and reducing oxidative damage, and simultaneously promoting cell proliferation and angiogenesis (10, 11).

In this research, we conducted vitrification on the ovarian cortex of domestic cats and added α-ALA during the vitrification process to explore its role in reducing damage caused by vitrification. Our findings indicated that α-ALA can mitigate oxidative stress damage and decrease the abnormality rate of primordial follicles. RNA-seq indicated that α-ALA addition enhanced the differences in cytoskeleton and chromosome separation induced by vitrification. By exploring the protective effects of α-ALA in cat ovarian vitrification, we gained theoretical insights of α-ALA in ovarian preservation and established a scientific foundation for clinical fertility preservation.

## 2 Materials and methods

### 2.1 Animals and experimental groups

Ovarian tissue samples were collected from domestic cats (7 months to 2 years old) after routine ovariohysterectomy at local veterinary clinics. The study was conducted with the approval of the Medical Ethics Committee of Yantai Yuhuangding Hospital (reference number 2025-168). The excised tissues were immediately placed in chilled phosphate-buffered saline (PBS) containing 100 IU/mL penicillin and 100 mg/mL streptomycin, and transported to the laboratory at 4°C within 12 h of excision. The ovarian cortices were sectioned into 2 × 2 ×1 mm-sized pieces with the following dissection medium: Hank’s MEM supplemented with 10 mM HEPES, 1 mM pyruvate, 2 mM L-glutamine, 1% (v/v) penicillin streptomycin solution, and 0.1% bovine serum albumin. The ovarian fragments were divided into four groups as follows: One group was fixed in 4% paraformaldehyde (PFA) as the fresh control, while the remaining three groups were exposed to vitrification solutions supplemented with 0, 100, or 150 μM α-ALA respectively, and then vitrified and stored in liquid nitrogen for at least 48 h before warming. We collected a total of 40 ovaries from 20 cats for experimental analysis. Each experiment was repeated more than three times. A total of 72 ovarian tissue fragments were employed in this research. Moreover, 45 ovarian fragments were not subjected to *in vitro* culture, among which nine fresh ovarian fragments were fixed for section preparation and used for hematoxylin and eosin (HE), immunohistochemistry (IHC), and TUNEL analysis, respectively; Another 27 ovarian fragments underwent vitrification with 0, 100, or 150 μM α-ALA treatments, respectively (nine per group), employed for HE, IHC, and TUNEL analysis. Additionally, three fresh ovarian fragments were employed for RNA-seq, and another six ovarian fragments were vitrified with 0 or 150 μM α-ALA treatments used for RNA-seq (three per group). A total of 27 ovarian fragments were used for *in vitro* culture, among which 9 fresh ovarian fragments were used for HE, IHC, and Masson staining, respectively, and another 18 ovarian fragments underwent vitrification with 0 or 150 μM α-ALA treatments (nine per group), correspondingly employed for HE, IHC, and Masson analysis, respectively.

### 2.2 Vitrification and warming

The domestic cat ovarian cortex vitrification method was based on published article (12) as follows: The cortical pieces were exposed to an equilibration solution (7.5% DMSO+ 7.5% EG+ 20% fetal bovine serum (FBS) in base medium) for 10 min at 4°C, moved the samples to a vitrification solution (15% DMSO + 15% EG + 20% FBS in base medium) for 10 min at 4 °C, then moved to metallic flakes, directly plunged into liquid nitrogen, and stored in liquid nitrogen. Warming was performed by removing the open metallic flakes carrier and immersing it in washing solutions (1, 0.5, 0.25, and 0 M sucrose in base medium) for 5 min at each step at 37°C, respectively.

### 2.3 Culture

Fragments were cultured in MEM medium supplemented with the following components: 50 μM ascorbic acid, 0.3% (w/v) polyvinyl alcohol, 1% (v/v) insulin-transferrin-selenium (ITS) mixture, 10 μg/mL follicle stimulating hormone (FSH), and 1% (v/v) penicillin-streptomycin solution. The α-ALA-supplemented group received additional supplementation with 150 μM α-ALA. All cultures were maintained at 37°C in a humidified atmosphere containing 5% CO_2_, with medium replacement performed every other day. The samples were collected on days 2 and 7 of culture.

### 2.4 Ovarian histomorphometric analysis

The samples were collected and fixed in 4% PFA for at least 24 h. Following dehydration and paraffin embedding, the samples were processed by sectioning (5 μm) and stained with HE. The paraffin-embedded ovarian sections were dewaxed (xylene I for 10 min, xylene II for 10 min) and rehydrated (100% alcohol I for 10 min, 100% alcohol II for 10 min, 95% alcohol I for 10 min, 95% alcohol II for 10 min, 80% alcohol for 10 min, 70% alcohol for 10 min), and then submerged in distilled water. The sections were then stained with hematoxylin and counterstained with eosin. After dehydration, the coverslips were mounted, and images were captured using a Leica Camera (Leica Microsystem, Buffalo Grove, IL, United States).

Follicles were graded as follows: The primordial follicle (grade 1) contained a spherical oocyte surrounded by a single layer of flattened granulosa cells. The primary follicle (grade 2) is characterized by the transformation of granulosa cells into a cuboidal shape, an increase in oocyte size, and the initial formation of the zona pellucida. The secondary follicle (grade 3) is defined by multiple layers of cuboidal granulosa cells, the appearance of theca cells, and the beginning accumulation of follicular fluid. The antral follicle (grade 4) is distinguished by the presence of a fluid-filled antral cavity, along with the formation of the umulous oophorus and a corona radiata.

### 2.5 IHC analysis

The protein expression levels in ovarian fragments were analyzed using IHC staining on the paraffin slides. The ovarian sections fixed in the paraffin underwent dewaxing and rehydration procedures, followed by sequential steps of antigen retrieval and endogenous peroxidase inactivation through incubation with 3% H_2_O_2_ for 10 min. Following blocking with 3% bovine serum albumin (BSA), the slides were incubated overnight with primary antibodies of anti-SOD1 (1:200, Proteintech, 10269-1-AP), anti-Caspase3 (1:200, Proteintech, 66470-2-Ig), and anti-Ki67 (1:200, Abcam, a15580) in a humidified box at 4°C. Following three PBS washes, sections were incubated with species-specific secondary antibodies (anti-rabbit IgG, 1:200, Affinity, S0001; anti-mouse IgG, 1:200, Affinity, S0001) at 37°C for 30 min. Afterward, the slides were stained with diaminobenzidine (DAB) (ZSGB-BIO, Beijing, China) as a chromogen, followed by hematoxylin counterstaining according to the manufacturer’s protocols. Finally, dehydrated sections were mounted with neutral balsam and imaged using a Leica Camera (Leica Microsystem, Buffalo Grove, IL, United States).

### 2.6 RNA extraction and real-time reverse transcription polymerase chain reaction

Total RNA was extracted from tissues using TRIzol reagent (Vazyme Biotech, Nanjing, China) based on the manufacturer’s instructions. Subsequently, cDNA synthesis was performed with HiScript III RT SuperMix for qPCR (+gDNA wiper) (Vazyme Biotech, Nanjing, China) under recommended reaction conditions. The qPCR analysis was conducted using Taq Pro Universal SYBR qPCR Master Mix (Vazyme Biotech, Nanjing, China). Gene expression levels were calculated using the comparative 2^−ΔΔCt^ method and presented as mean fold changes ± standard deviation (s.d.). The primer sequences used are provided in **Supplementary Table 1**.

### 2.7 RNA-seq data processing and analysis

#### 2.7.1 RNA-seq data analysis

Raw RNA-seq reads were quality-checked and trimmed using fastp (v0.23.2) with default parameters to remove adapters and low-quality bases. Trimmed reads were aligned to the reference genome using HISAT2 (v2.2.1) with default settings for paired-end reads, ensuring high alignment accuracy. Gene counts were quantified using featureCounts (v2.0.1) from the Subread package, with annotations from the reference genome GTF file, counting only uniquely mapped reads. Gene expression levels were normalized as FPKM (fragments per kilobase of exon per million mapped reads) using StringTie (v2.1.5) to enable comparison across samples.

Differential gene expression analysis was conducted using DESeq2 (v1.36.0) in R, based on raw read counts from featureCounts. Genes were considered differentially expressed if they exhibited an absolute log2 fold change (|log2(FoldChange)|) ≥ 1 and an adjusted p-value (padj) ≤ 0.05, corrected for multiple testing using the Benjamini-Hochberg method. Functional enrichment analysis of differentially expressed genes (DEGs) was performed using clusterProfiler (v4.0.5), assessing Gene Ontology (GO) terms and KEGG pathways with a significance threshold of padj ≤ 0.05.

#### 2.7.2 Principal component analysis

To evaluate sample variability, Principal Component Analysis (PCA) was performed using FPKM-normalized gene expression values. Data were log2-transformed and scaled to unit variance to ensure equal contribution of genes. The covariance matrix was computed, followed by eigenvalue and eigenvector decomposition using R’s prcomp function. The first two principal components, accounting for 65.05% of the total variance, were selected for visualization. PCA plots were generated using ggplot2 (v3.3.6) in R, with samples color-coded by experimental condition to highlight clustering patterns.

### 2.8 Statistical analysis

Statistical significance was analyzed by one-way ANOVA or two-way ANOVA with Tukey’s multiple comparisons test (*P < *0.05 was considered statistically significant). For non-normally distributed data, non-parametric Kruskal–Wallis test with Dunn’s multiple comparisons test was performed. Unless otherwise specified, experiments were independently replicated three times with biological replicates. The data are presented as the mean ± s.d. Significance levels are denoted as follows: *P < 0.05; **P < 0.01.

## 3 Results

### 3.1 α-ALA alleviated vitrification-induced follicular morphological abnormalities

**Figure 1:**
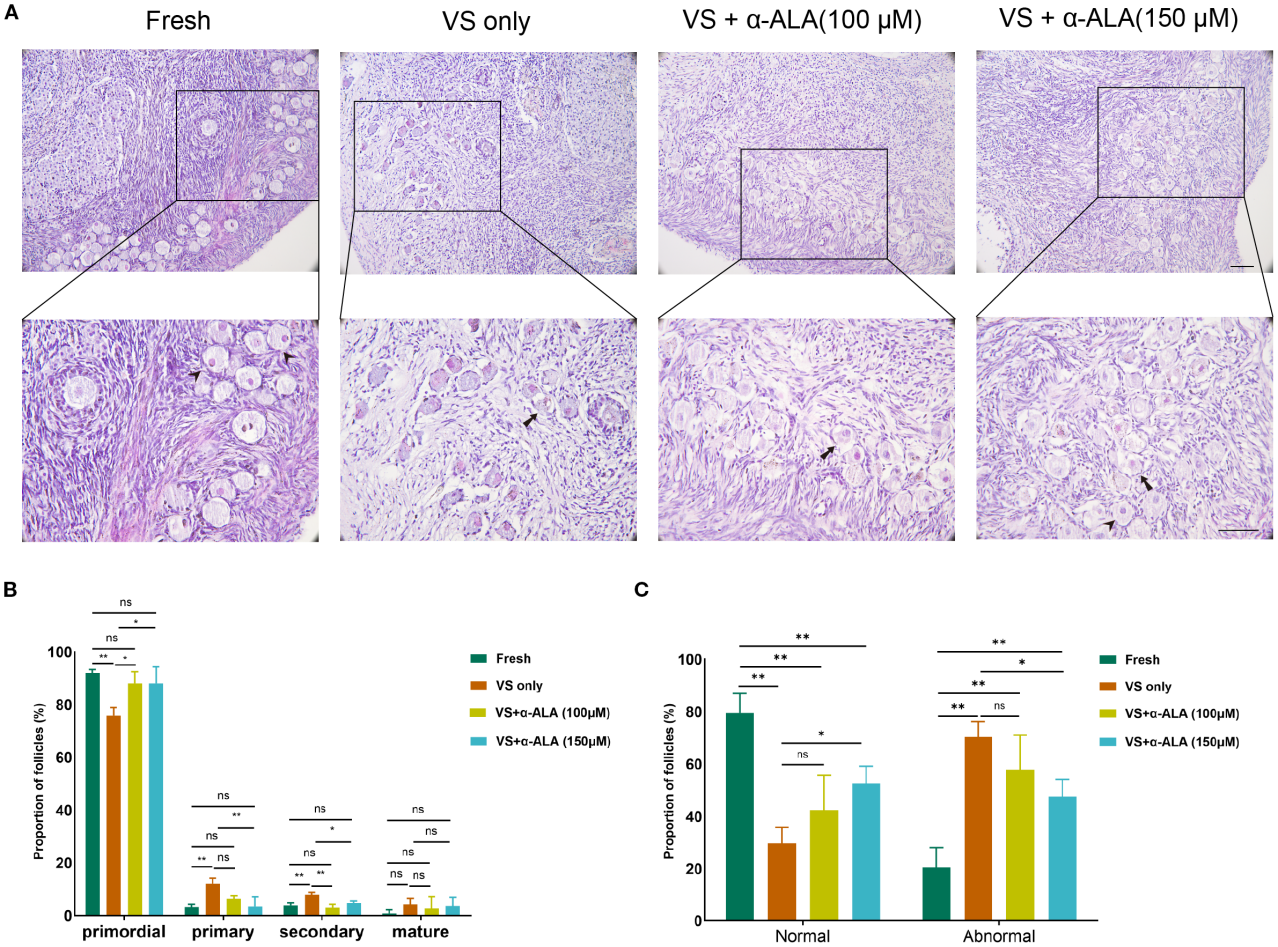
α-ALA alleviated vitrification-induced follicular morphological abnormalities. **(A)** HE staining of ovarian cortex in Fresh, VS only, VS + α-ALA (100 μM), and VS + α-ALA (150 μM) groups. (Arrows indicate normal follicles, and arrowheads indicate abnormal follicles). **(B)** Follicle proportions of primordial, primary, secondary and mature follicles in various groups. **(C)** Normal and abnormal follicle proportions in different groups (n = 3, scale bar = 100 μm. *P < 0.05, **P < 0.01).

To quantified the numbers of primordial follicles and developing follicles, we calculated the incidence of abnormal follicles in fresh and vitrified-thawed ovarian groups. As indicated in **Figures 1A, B**, vitrification resulted in a significant decrease in the proportion of primordial follicles. While the proportions of primary and secondary follicles indicated some increase, this may be attributed to the significant loss of primordial follicles. On the other hand, vitrification significantly increased the incidence of follicular abnormalities. Although the proportion of follicular abnormalities in the α-ALA-supplemented group remained higher compared to the Fresh group, it revealed a significant decrease relative to the VS only group (**Figure 1C**). This indicated that α-ALA can partially ameliorate follicular abnormalities during the vitrification process.

### 3.2 α-ALA reduced cell apoptosis induced by vitrification

**Figure 2:**
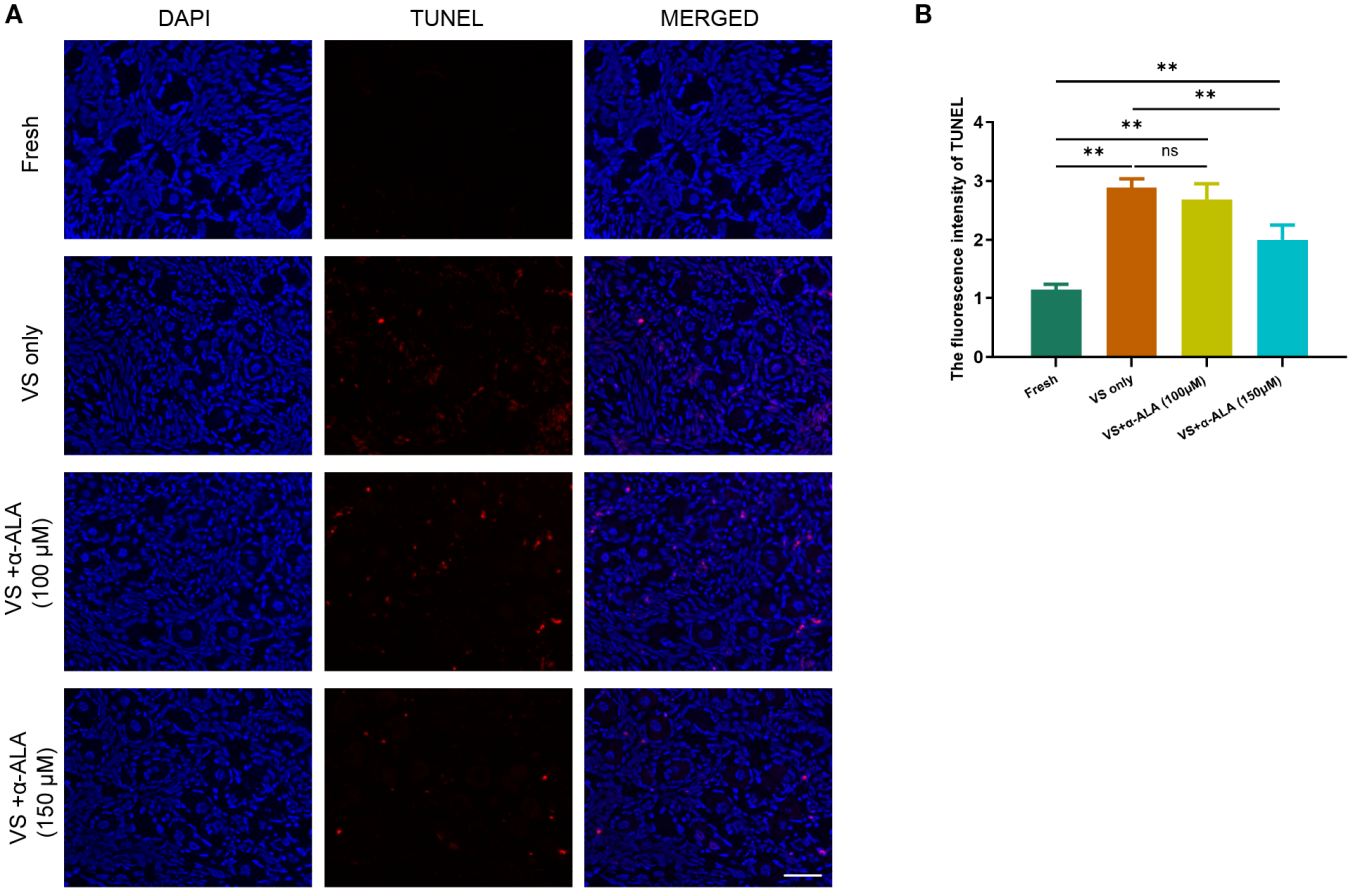
α-ALA reduced vitrification-induced TUNEL apoptosis. **(A)** TUNEL staining of ovarian cortex in Fresh, VS only, VS + α-ALA (100 μM) and VS + α-ALA (150 μM) groups. (red fluorescence: TUNEL; blue fluorescence: DAPI). **(B)** Statistical diagram of the average fluorescence intensity of TUNEL in different groups (n = 3, scale bar = 50 μm. **P < 0.01).

**Figure 3:**
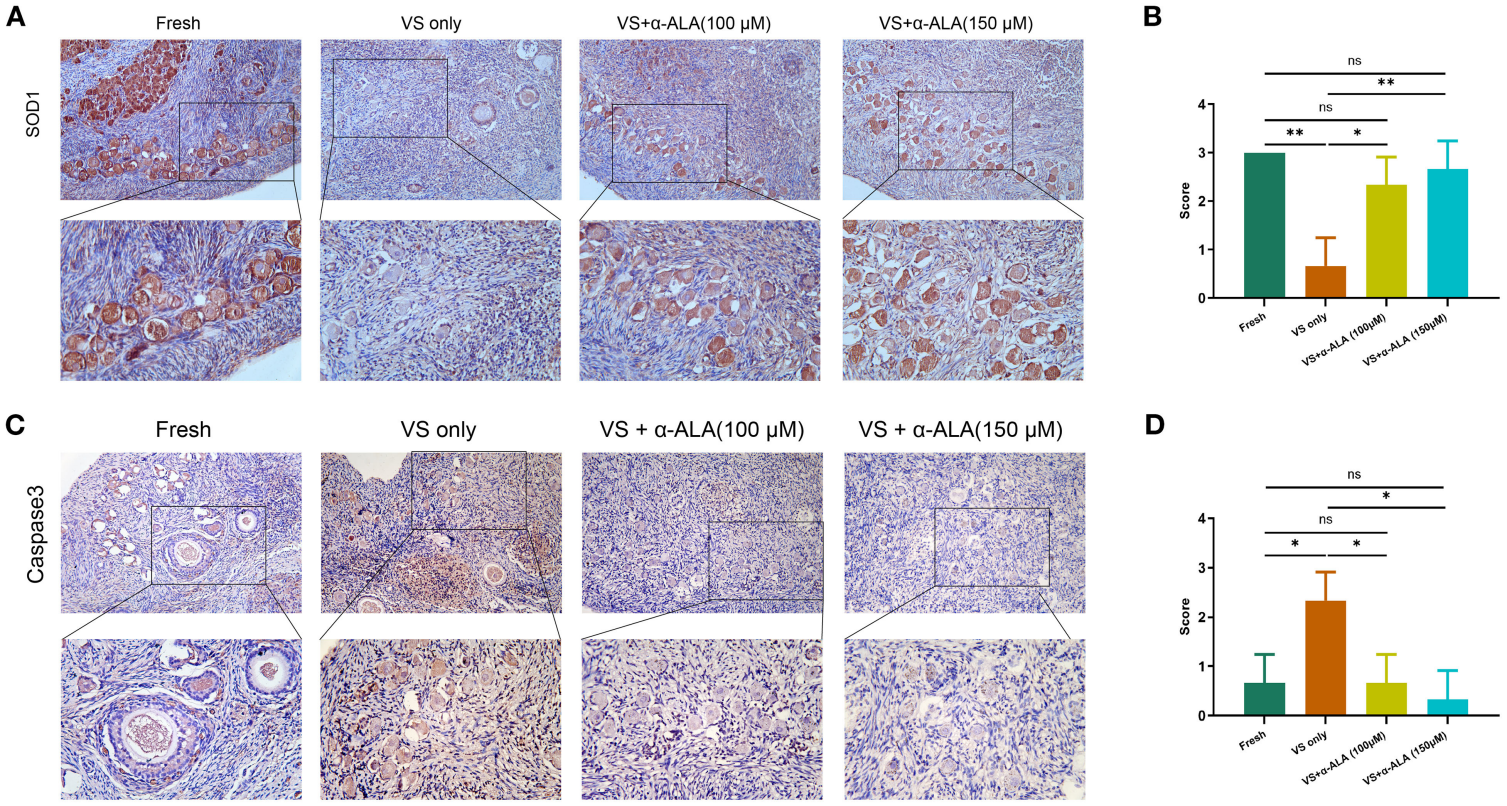
IHC staining of apoptosis-related proteins in ovarian cortex. **(A)** IHC staining and **(B)** quantifications of SOD1. **(C)** IHC staining and **(D)** quantifications of caspase3 (n = 3, scale bar = 100 μm. *P < 0.05, **P < 0.01).

We performed TUNEL staining analysis to validate the protective role of α-ALA. The results revealed that supplementation with 150 μM α-ALA significantly decreased TUNEL fluorescence intensity compared with the VS only group (**Figures 2A, B**). Subsequently, we performed IHC analysis of the anti-apoptotic protein SOD1 and the pro-apoptotic protein Caspase3. The results indicated that vitrification led to a significant decrease in SOD1 expression, which was effectively counteracted by α-ALA at 100 and 150 μM concentrations (**Figures 3A, B**). Concurrently, α-ALA could also reduce the abnormal upregulation of caspase3 caused by vitrification, and both 100 and 150 μM concentrations effectively reduced its levels (**Figures 3C, D**). Based on the above results, we concluded that α-ALA ameliorates vitrification-induced cell apoptosis, and we selected a concentration of 150 μM for subsequent studies.

### 3.3 Effects of α-ALA on tissue *in vitro* culture

**Figure 4:**
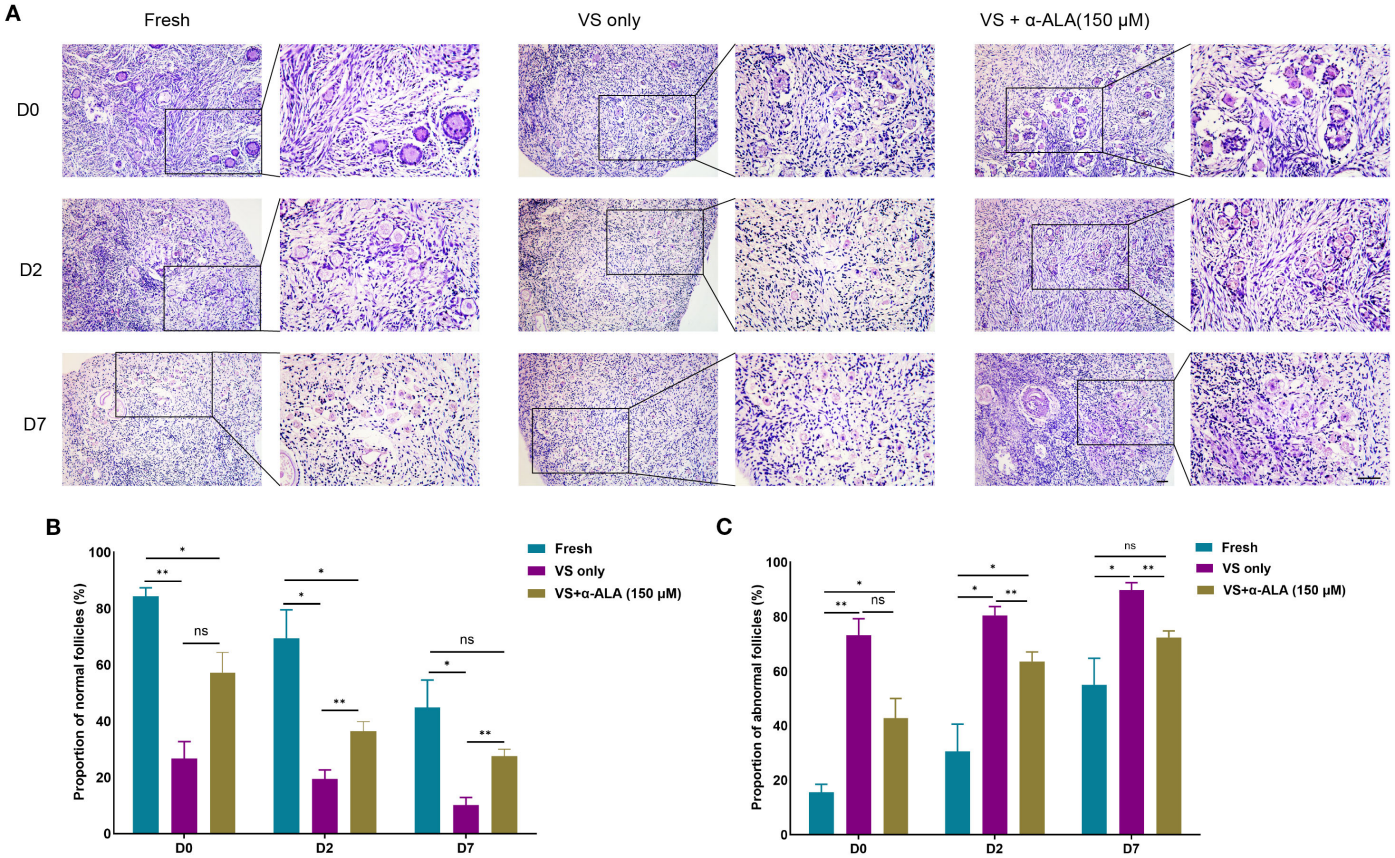
Effects of α-ALA on tissue *in vitro* culture. **(A)** HE staining of ovarian cortex *in vitro* cultured for days 0, 2, and D7 in Fresh, VS only, and VS + α-ALA (150 μM) groups. **(B)** Normal follicle proportions in different groups. **(C)** Abnormal follicle proportions in different groups (n = 3, scale bar = 50 μm. *P < 0.05, **P < 0.01).

We cultured ovarian cortex tissues from Fresh, VS only, and VS + α-ALA (150 μM) groups *in vitro*, and quantified the proportions of normal and abnormal follicles in each groups. The results demonstrated that the VS only group exhibited a higher number of abnormal follicles than the other two groups at days 2 and 7, while supplementation with 150 μM α-ALA mitigated the vitrification-induced follicular abnormality relative to the VS only group (**Figure 4**).

### 3.4 α-ALA attenuated apoptosis during *in vitro* culture of ovarian cortex tissue

**Figure 5:**
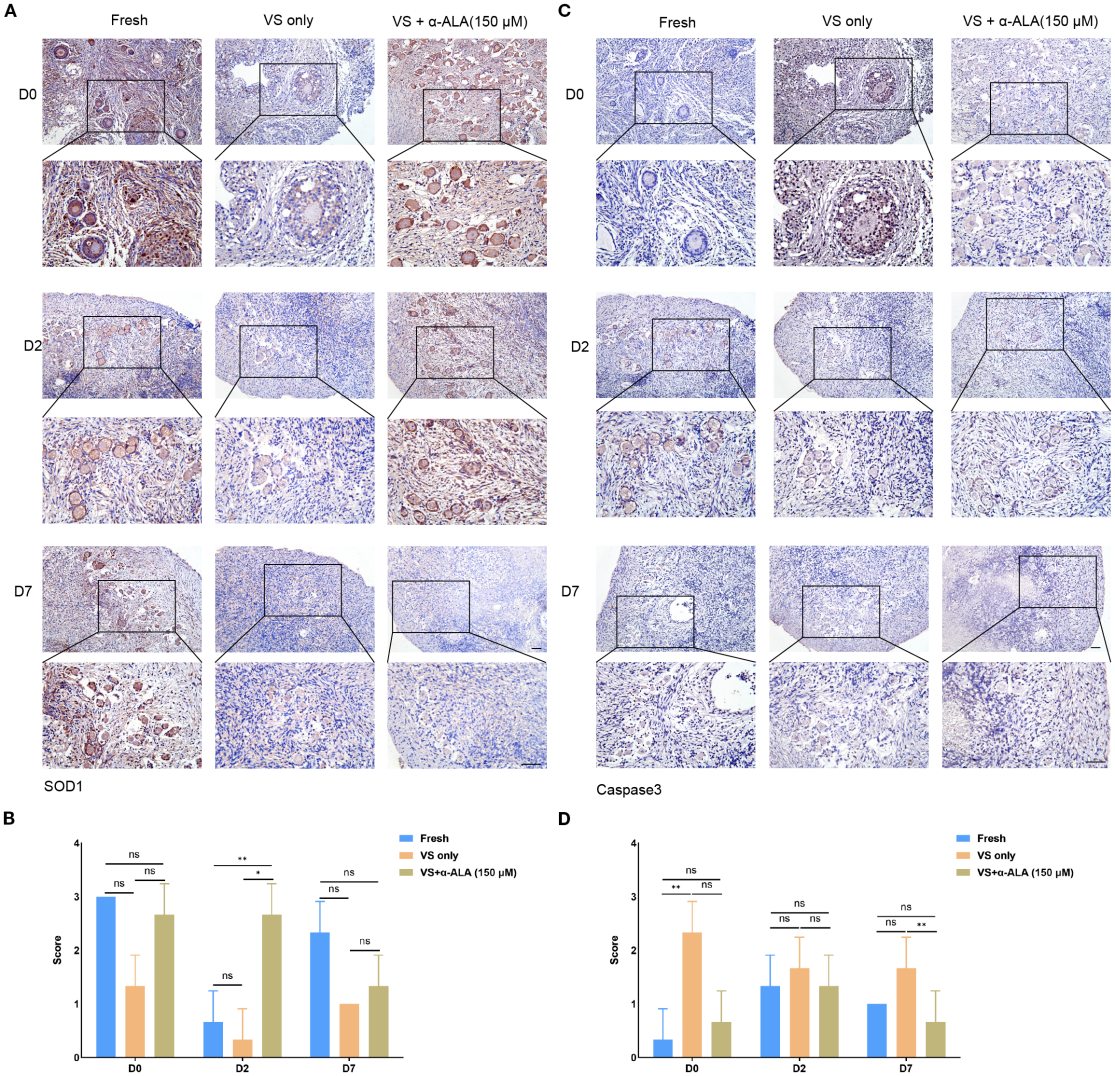
IHC staining of apoptosis-related proteins in *in vitro* cultured ovarian cortex tissues. **(A)** IHC staining and **(B)** quantifications of SOD1 in *in vitro* cultured ovarian cortex tissues at days 0, 2, and 7. **(C)** IHC staining and **(D)** quantifications of caspase3 in *in vitro* cultured ovarian cortex tissues at days 0, 2, and 7 (n = 3, scale bar = 100 μm. *P < 0.05, **P < 0.01).

**Figure 6:**
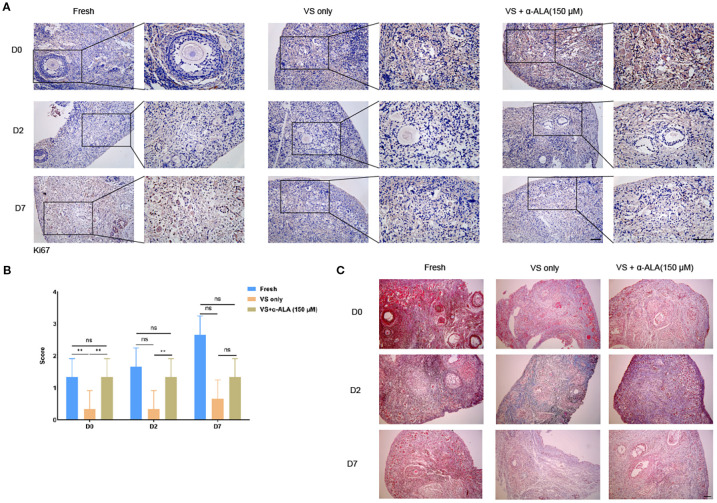
Masson staining of *in vitro* cultured ovarian cortex tissues. **(A)** IHC staining and **(B)** quantifications of Ki67 in *in vitro* cultured ovarian cortex tissues at days 0, 2, and 7 (n = 3, scale bar = 50 μm. **P < 0.01). **(C)** Masson staining of *in vitro* cultured ovarian cortex tissues at days 0, 2, and 7. (Scale bar = 200 μm).

We analyzed apoptosis process of the *in vitro* culture ovarian cortex. The results indicated that α-ALA treatment significantly enhanced SOD1 expression levels by day 2 compared with the VS only group (**Figures 5A, B**). Caspase3 expression was down-regulated at day 7 in the VS + α-ALA (150 μM) group compared with the VS only group (**Figures 5C, D**). Apoptosis may lead to fibrosis. Masson staining results suggested that α-ALA can delay fibrosis development, particularly at two days of culture (**Figure 6C**). Moreover, Ki67 staining indicated that after two days of culture, Ki67 expression in the VS + α-ALA (150 μM) group was increased compared with the VS only group (**Figures 6A, B**). Based on the above results, we concluded that α-ALA can alleviate apoptosis induced by vitrification and ameliorate the fibrosis process.

### 3.5 Differential gene expression in Fresh, VS only, and VS + α-ALA groups

**Figure 7:**
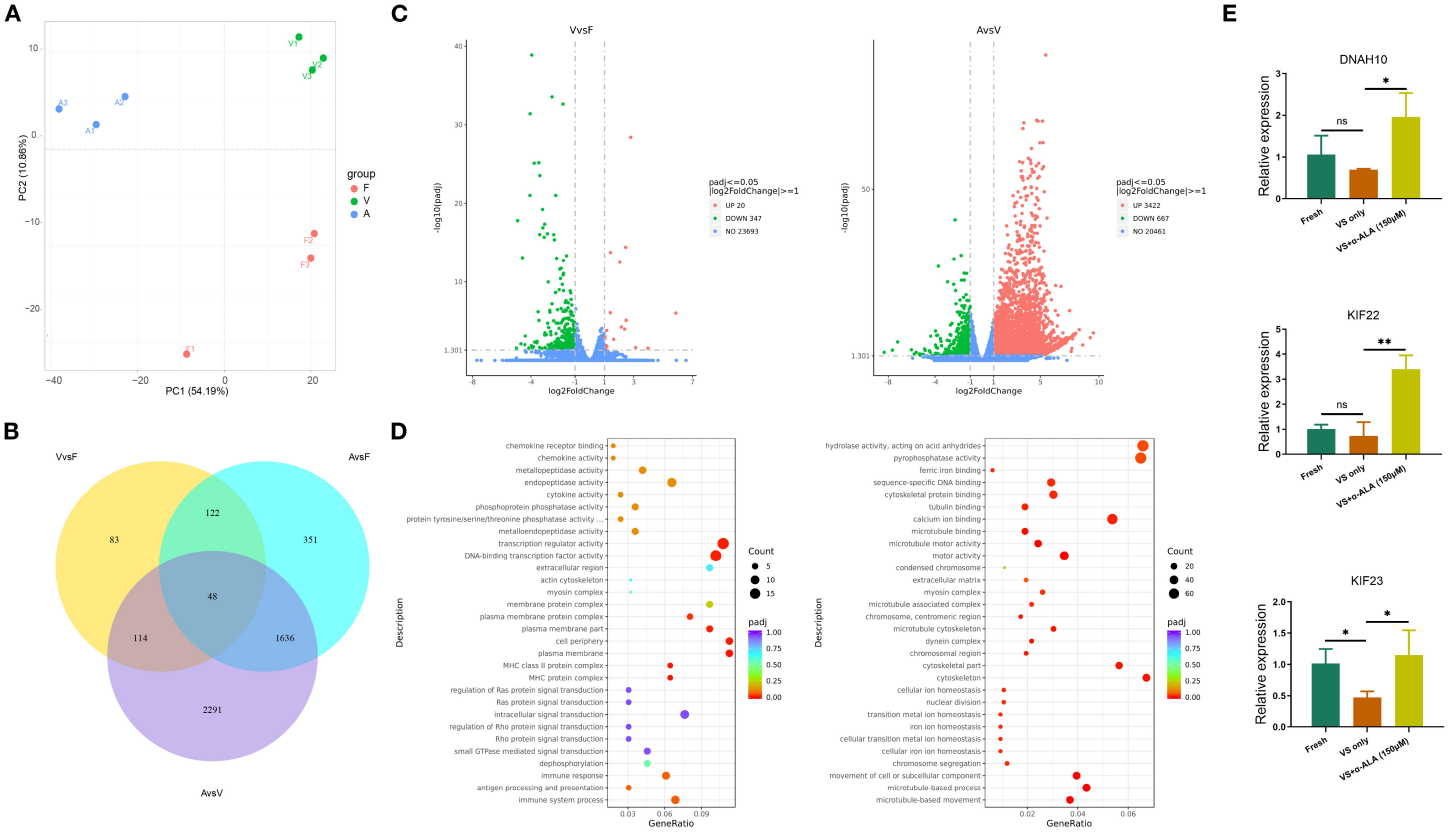
Differential gene expression analysis and DEGs functional enrichment analysis. **(A)** PCA analysis of Fresh, VS only, and VS + α-ALA (150 μM) groups. **(B)** Venn diagram indicating the intersection of co-expressed genes and DEGs. **(C)** Scatter-plot comparison revealing the DGEs between Fresh and VS only groups, VS only and VS + α-ALA (150 μM) groups. **(D)** Bubble diagram demonstrating the DEGs functional enrichment analysis between Fresh and VS only groups, VS only and VS + α-ALA (150 μM) groups. **(E)** The qRT-PCR verification of DNAH10, KIF22, and KIF23 (n=3, *P < 0.05, **P < 0.01).

RNA-seq data showed that approximately 36–45 million reads were successfully mapped to the genome for each sample (**Supplementary Table 2**). PCA revealed distinct clustering patterns among Fresh, VS only, and VS + α-ALA groups (**Figure 7A**). The Venn diagram illustrated genes uniquely expressed in individual groups and those co-expressed between different groups (**Figure 7B**). Comparative analysis ofDEGs was conducted across the different groups. As indicated in **Figure 7C**, a total of 367 DEGs were detected between VS only and Fresh groups: 20 upregulated genes and 347 downregulated genes. Moreover, we compared DEGs in VS + α-ALA and VS only groups, and identified that 3422 genes were upregulated and 667 genes were downregulated.

### 3.6 GO term and KEGG pathway enrichment analysis of DEGs and qRT-PCR validation

We performed enrichment analysis of gene functions based on DEGs, enriched GO terms, and KEGG pathways. Functional enrichment analysis of downregulated genes in the VS only group compared with the Fresh group revealed enrichment in the actin cytoskeleton and extracellular region. Subsequently, we verified the upregulated signals following α-ALA addition compared to the VS only group. The result showed that extracellular matrix, chromosome separation, and multiple regulatory pathways associated with the cytoskeleton were enriched, including cytoskeletal protein binding, microtubule motor activity, and cytoskeleton (**Figure 7D**). To verify the transcriptome sequencing results, we performed qRT-PCR. The results revealed that KIF23 expression was downregulated in the VS only group, while its expression was rescued in the VS + α-ALA (150 μM) group. Additionally, KIF22 and DNAH10 expression levels were upregulated following α-ALA treatment compared to the VS only group (**Figure 7E**).

## 4 Discussion

The primary challenges during ovarian tissue vitrification are ROS-induced oxidative stress, which can trigger a cascade of detrimental effects, including aberrant follicular morphology, disrupted follicular development, apoptosis, post-transplant endocrine dysfunction, and compromised fertility restoration (10, 13). α-ALA exhibits antioxidant activity by directly scavenging ROS and synergizing with the antioxidant network, thereby indirectly contributing to the recycling of other intracellular antioxidants (14, 15). In this study, we supplemented α-ALA during cat ovarian cortex vitrification to investigate its anti-apoptotic effects and other impacts during the process.

Ovarian tissue comprises various cell types, each requiring distinct vitrification conditions, which makes tissue cryopreservation more complex than the vitrification of oocytes or embryos (16, 17). Within the ovarian cortex, there exists an abundant reserve of primordial follicles, whose quantity and quality are intimately associated with ovarian reserve. Consequently, the central objective of ovarian tissue vitrification is to maintain the structural and functional integrity of primordial follicles. Moreover, researchers have explored various concentrations of cryoprotectants and added antioxidants to attenuate follicle damage caused by vitrification. In the study, we quantified the proportions of normal and abnormal follicles, as well as primordial and developing follicles. The results demonstrated that α-ALA ameliorated follicular abnormalities induced by vitrification, with the 150 μM treatment group indicating a significant improvement (**Figure 1**). These findings indicated that α-ALA improved the quality of vitrified ovarian tissue. Research has indicated that α-ALA partially rescued follicle loss in glutathione-deficient mice and restored ovarian function, demonstrating its role in the recovery of ovarian reserve (18). Since morphological analysis by HE staining can only reflect the quality of ovarian tissue and follicles to a certain extent, but can not determine the degree of cell apoptosis, we performed TUNEL staining. The results revealed that the VS + α-ALA (150 μM) group exhibited significantly lower cell apoptosis compared to the VS only group, indicating that α-ALA can reduce apoptosis. Mottola et al. found that 200 μM α-ALA decreases H_2_O_2_-induced DNA damage in human amniotic cells (19). Li et al. discovered that α-ALA decreased oxidative stress and apoptosis in human corneal epithelial cells caused by a high-glucose environment (20). Studies have validated that α-ALA possesses potent antioxidant capabilities, which can enhance glutathione levels and ameliorate oxidative stress in various cell types (21).

The caspase family serves as a central regulator of cell apoptosis. Caspase3 functions as the primary executioner enzyme, coordinating the final stages of apoptosis through the targeted cleavage of its substrates. During the terminal phase of apoptosis, caspase3 specifically cleaves various caspase substrates, exerting a decisive function that leads to the disintegration of cellular structures and the collapse of DNA repair mechanisms, ultimately driving the completion of the apoptotic program (22, 23). Gorricho et al. investigated the size of cat ovarian tissue fragments during vitrification and identified the appropriate size by assessing follicle morphology and apoptosis, including differences in caspase3 expression (24). To further validate the anti-apoptotic effect of α-ALA during vitrification, we conducted caspase3 IHC analysis. The results revealed a downregulation of caspase3 in VS + α-ALA (150 μM) group, demonstrating the anti-apoptotic effect of α-ALA (**Figure 3B**). Moreover, we performed SOD1 IHC analysis. SOD1 is a critical antioxidant enzyme that possesses antioxidant activity, scavenging superoxide radicals and effectively reducing ROS accumulation, thereby protecting cells from oxidative damage (25). Low-level oxidative stress is essential for maintaining primordial follicle dormancy, and the high expression of SOD1 in oocytes counteracts ROS accumulation (26). Studies have indicated that administering antioxidant treatment during oocyte or embryo development, which enhances the activity of antioxidant enzymes, such as SOD1, can improve the quality of oocytes and embryos (27, 28).

α-ALA attenuated the apoptosis process and facilitated the restoration of cell proliferation capacity during the culture. Studies have indicated that supplementation of antioxidants and growth factors during the *in vitro* culture of vitrified-warmed ovarian tissue enhanced the expression of antioxidant genes such as SOD1, promoted the activation of primordial follicles, and stimulated follicular development (29, 30). α-ALA addition during the *in vitro* culture of bovine secondary preantral follicles reduced the expression of apoptosis-related genes and promoted follicles growth and development (31). Our study also verified the anti-apoptotic effect of α-ALA (**Figure 5**). Meanwhile, fibrosis may occur during the *in vitro* culture process of vitrified-thawed tissue. Our results indicated that the VS only group exhibited higher fibrosis levels than the VS + α-ALA(150 μM) group on day 2 of culture (**Figure 6**). Ovarian cortex vitrification and thawing, ischemia-reperfusion injury, and inflammation may all lead to fibrosis. Fibrosis occurring in the ovarian cortex may further impair the function of stromal cells, thereby affecting primordial follicles dormancy and follicular development (32, 33). α-ALA application during ovine ovarian vitrification mitigated vitrification-induced aging and fibrosis (11). Our results align with the conclusions of the aforementioned studies.

Our transcriptome sequencing revealed alterations in transcription regulator activity, actin cytoskeleton, myosin complexes, and extracellular regions between the VS only and Fresh groups. Furthermore, we enriched the rescued signaling pathways following α-ALA supplementation, including sequence-specific DNA binding, myosin complexes, cytoskeleton organization, microtubule cytoskeleton, chromosome separation and extracellular matrix (**Figure 7**). Transcriptome sequencing demonstrated that vitrification of mouse ovarian tissue resulted in changes in cellular membranes, cytoskeletons, and extracellular matrices (7). Moreover, studies have confirmed that mouse follicles vitrification leads to DNA fragmentation, alterations in oocyte-granulosa cell apposition, and changes in actin (34). Oocyte vitrification may also lead to damage in the cytoskeleton and spindle to some extent, as observed in the vitrification of mouse, cat, and cattle oocytes (35–37).

In this study, we focused on the anti-apoptotic effects of α-ALA in cat ovarian cortex vitrification, and evaluated the impact of vitrification on the cytoskeleton using transcriptomic analysis. Subsequent research should focus more closely on the impact on follicular development.

## 5 Conclusion

In summary, our results demonstrated that 150 μM α-ALA alleviated oxidative stress induced by vitrification, enhanced antioxidant capacity during *in vitro* culture, and thereby partially preserved cell proliferation capacity and delayed the onset of fibrosis. Additionally, our analysis revealed the differential expression patterns of transcription factors and signaling pathways between fresh cat ovarian cortex and the vitrified tissue, as well as the effects of α-ALA on vitrification at the transcriptomic level.

## Data Availability

The original contributions presented in the study are included in the article/**Supplementary Material**. Further inquiries can be directed to the corresponding authors.
