# Supplementary material for: Alpha-lipoic acid reduces oxidative damage and ameliorates follicular abnormalities in vitrified cat ovarian tissue
Source: Front Endocrinol (Lausanne). 2025 Oct 13;16:1682526. doi: 10.3389/fendo.2025.1682526 (PMC12554562; doi:10.3389/fendo.2025.1682526)
Supplement: Supplementary file 1 [file Table1.docx]

**Supplementary Table 1 Primer for qRT-PCR**

| Gene names | primers (5’-3’) | Product size (bp) |  |
| --- | --- | --- | --- |
| *Gapdh* | F: CAACTGCTTAGCACCCCTGGCC  R: CTGACACGTTGGCAGTGGGGAC | 253 |  |
| *DNAH10* | F: AAAGTGAGCCCGGCGTGATGTG  R: AGAATCGGCAGGTCGACCACCA | 280 |  |
| *KIF22* | F: GAGCAGGCGGCATCCCCAAA  R: GGCCAATGCGTTGCAGGCTG | 220 |  |
| *KIF23* | F: CAGCACCTGCCCAACCAGATGG  R: GGGTCCCAGCTGAGATCCTGCT | 101 |  |
