# Supplementary material for: Alpha-lipoic acid reduces oxidative damage and ameliorates follicular abnormalities in vitrified cat ovarian tissue
Source: Front Endocrinol (Lausanne). 2025 Oct 13;16:1682526. doi: 10.3389/fendo.2025.1682526 (PMC12554562; doi:10.3389/fendo.2025.1682526)
Supplement: Supplementary file 2 [file Table2.docx]

**Supplementary Table 2 Alignment statistics of reads align to the reference genome.**

| **Name** | **Total_reads** | **Total_map** | **Unique_map** | **Splice_map** | | **Unsplice_map** | **Proper_map** |
| --- | --- | --- | --- | --- | --- | --- | --- |
| **F1** | 41569766 | 37590170  (90.43%) | 35653247  (85.77%) | 11557831(27.8%) | 24095416  (57.96%) | | 32624292  (78.48%) |
| **F2** | 46376458 | 42639375  (91.94%) | 39668729  (85.54%) | 13118579(28.29%) | 26550150  (57.25%) | | 36880400  (79.52%) |
| **F3** | 39465106 | 36271439  (91.91%) | 33975884  (86.09%) | 12549100(31.8%) | 21426784  (54.29%) | | 31132566  (78.89%) |
| **V1** | 44117462 | 41572630  (94.23%) | 35816936  (81.19%) | 12693273(28.77%) | 23123663  (52.41%) | | 34057606  (77.2%) |
| **V2** | 47052006 | 42841828  (91.05%) | 36987156  (78.61%) | 12378012(26.31%) | 24609144  (52.3%) | | 33427080  (71.04%) |
| **V3** | 49013344 | 45246146  (92.31%) | 39394829  (80.38%) | 13693836(27.94%) | 25700993  (52.44%) | | 36675462  (74.83%) |
| **A1** | 47992166 | 43794452  (91.25%) | 40025719  (83.4%) | 14556780(30.33%) | 25468939  (53.07%) | | 35944240  (74.9%) |
| **A2** | 42092192 | 38681097  (91.9%) | 32319405  (76.78%) | 12423315(29.51%) | 19896090  (47.27%) | | 29379826  (69.8%) |
| **A3** | 46866242 | 42737804  (91.19%) | 38589998  (82.34%) | 14292409(30.5%) | 24297589  (51.84%) | | 34968374  (74.61%) |
